# Potential of UK and US newspapers for shaping patients' knowledge and perceptions about antidiabetic medicines: a content analysis

**DOI:** 10.1186/s40545-022-00462-8

**Published:** 2022-10-15

**Authors:** Nadia Farhanah Syafhan, Gaoyun Chen, Carole Parsons, James C. McElnay

**Affiliations:** 1 Department of Clinical Pharmacy and Social Pharmacy, Faculty of Pharmacy, Universitas Indonesia, Depok, Indonesia; 2 Clinical and Practice Research Group, School of Pharmacy, Queen’s University Belfast, Belfast, BT9 7BL UK

**Keywords:** Antidiabetic medicines, Newspaper coverage, Newspaper portrayal

## Abstract

**Background:**

Information about how newspapers portray antidiabetic medicines to readers is lacking. This study investigated the reporting on antidiabetic medicines in the most widely circulated newspapers published in the United Kingdom (UK) and the United States (US) over a 10-year period.

**Methods:**

The Nexis UK database was used to identify and select relevant articles. Systematic content analysis of the articles which met the inclusion criteria (articles of any format that contained reference to antidiabetic medicines) within the highest circulated newspapers in the UK and US between 2009 and 2018 was conducted. Inter-rater reliability of coding was established using a 10% sample of the identified articles.

**Results:**

A total of 560 (369 UK and 191 US) relevant newspaper articles were retrieved. In the UK, the number of relevant articles showed a slightly increasing trend over the study period, while in the US, article numbers declined over the study period. Safety/risk of antidiabetic medicines was the most frequent theme covered by the articles (34.6%). Over one-third of the newspaper articles were written from a clinical perspective (37.7%). Insulin was the most commonly discussed class of antidiabetic medicine (23.1%). Control of blood sugar levels (53.1%) and side effects/toxicity (92.7%) were the most frequently reported benefit and risk of antidiabetic medicines, respectively. The most frequently reported organ systems harmed by antidiabetic medicines were the cardiovascular, endocrine and gastrointestinal systems. The UK newspapers were more likely to report the benefits of antidiabetic medicines (*p* = 0.005), while the US articles were more likely to report on harms/risks (*p* = 0.001). The majority of relevant articles (91.8%) were judged as having a balanced judgement, while 8.2% of the articles were rated as exaggerated.

**Conclusions:**

This study has revealed that antidiabetic medicines are indeed reported on by UK and US newspapers. As media portrayal has the potential to negatively or positively influence patients’ views of their medication for diabetes, healthcare professionals should check on patients’ beliefs and knowledge about their medication and proactively provide objective and balanced information (including promotion of medication adherence).

## Background

The prevalence of diabetes mellitus worldwide among adults has risen over recent decades, and it has been estimated that there will be more than 600 million diabetes patients globally by 2040 [1, 2]. In the United Kingdom (UK), it was reported that 3.8 million people had a diagnosis of diabetes in 2018. This figure has more than doubled in the two decades since 1998 [3]. A high prevalence of diabetes has also been reported in the United States (US), where there were more than 30 million people living with the condition in 2017 [4].

A controlled blood glucose level is an essential treatment target for a diabetic patient. Medication, diet and exercise are the most common interventions to manage diabetes and to prevent its complications [5]. Twenty years ago, available classes of antidiabetic medicines were limited to insulin, biguanides and sulfonylureas, but in the last decade, newer insulin derivatives and classes of antidiabetic medicines have been approved for the treatment of diabetes. These include glucagon-like peptide-1 receptor agonists (GLP-1RA), dipeptidyl peptidase-4 inhibitors (DPP-4i) and sodium glucose transporter-2 inhibitors (SGLT2-i) [6].

Treatment guidelines for managing blood glucose in patients with diabetes, involving a wide range of antidiabetic medicines, are well established in the US and the UK [7, 8]. Knowledge and understanding of antidiabetic medicines plays an important role in the achievement of glycaemic control by people with diabetes [9, 10]. A number of published studies, including systematic reviews, have highlighted the high demand and requirement for good quality information by the growing diabetes population [11–14]. A recent multi-centre survey involving diabetic patients found that besides receiving information from their healthcare professionals, this population passively received health and medical information from traditional media, including newspapers. Traditional media outlets were used more frequently by this group of patients when compared with digital media, such as the internet [15]. Others have also found that newspapers remain one of the common sources of healthcare information for all chronic conditions, including diabetes [11, 15, 16].

In terms of content, newspapers are considered equivalent to other forms of news media. Whichever news media outlet broadcasts a particular piece of news first, other media outlets often pick up on the story quickly and broadcast it to the public, although the emphasis may be different across the different mass media outlets [17–20]. Available results from a 2015 survey found that 94% of adults in the UK read national printed or online newspapers at least once per month [21]. A 2018 survey also found that newspapers were listed as one of the main platforms used for news acquisition by adults in the UK [22]. There is no doubt that newspapers contribute to public understanding and perceptions relating to health-related issues [23–26]. The mass media, including newspapers, has been shown to impact on health-related behavioural change, for instance, in relation to alcohol use, smoking, cancer awareness, obesity, immunisation and AIDS [25–30].

Due to the importance of antidiabetic medicines in clinical practice and the potential of newspapers to be a highly influential vehicle for shaping knowledge and perceptions of diabetic patients about their medication, it was considered important to evaluate how antidiabetic pharmacotherapy is portrayed in the newspaper media.

## Methods

**Fig. 1:**
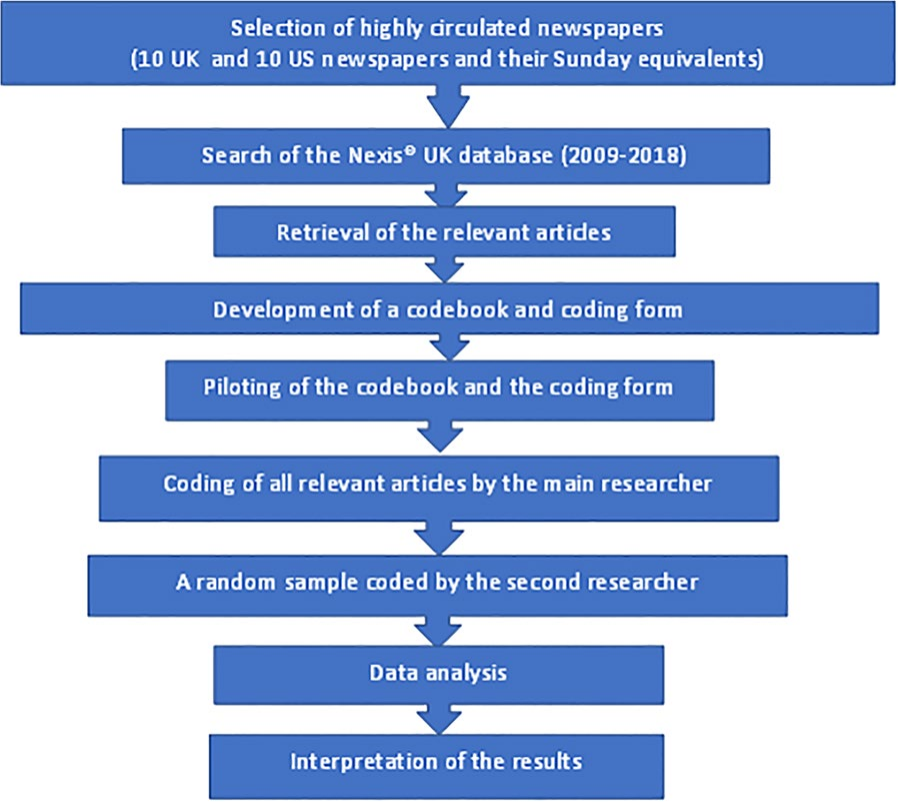
Overview of content analysis methodology

The aim of the present study was to investigate, through systematic content analysis, what information has been communicated to the general public about antidiabetic medicines via published newspaper articles in the UK and the United States (US) over a 10-year period (between 1st January 2009 and 31st December 2018) in the 10 highest circulated newspapers in the respective countries. An overview of the content analysis methodology used in the present study is illustrated in Fig. 1.

### Newspaper selection

The Nexis® UK database was used to search for electronically archived UK and US newspaper articles. A purposive sample of the 10 daily UK newspapers and their Sunday equivalents that had the highest circulation figures, according to the Audit Bureau of Circulations Ltd (ABC) [31], at the commencement of the study (March 2019) was selected. The titles included were *Metro, The Sun (The Sun on Sunday), Daily Mail (The Mail on Sunday), Evening Standard, Daily Mirror (Sunday Mirror), The Times (The Sunday Times), The Daily Telegraph (The Sunday Telegraph), Daily Star (Daily Star Sunday), Daily Express (Sunday Express)* and *the i*.

Using the same approach, a purposive sample of the top 10 daily US newspapers (and their Sunday equivalents) with the highest average circulation, and available on The Nexis® UK database, as ranked by the Alliance for Audited Media (AAM) [32], was also selected. This comprised *USA Today, The Wall Street Journal, The New York Times, Los Angeles Times, New York Post, Star Tribune, The Washington Post, Philadelphia Inquirer, Tampa Bay Times* and *Daily News*. All the US newspapers selected publish daily and also on Sundays, (except USA today and the Wall Street Journal). At the time the study was being conducted, several highly circulated US newspapers, including *Newsday, Chicago Tribune* and *The Boston Globe*, were not available in the Nexis® UK database and were, therefore, not included in the study.

### Search strategy, inclusion, and exclusion criteria

After carrying out empiric testing using various search terms, the search terms selected were “medication” OR “medicine” OR “drug” OR “treatment” OR “therapy” AND “diabet!”. The “diabet!” search term encompassed diabetes and diabetic(s). These broad search terms were applied to minimise loss of relevant articles.

To compare the trends in publication of relevant newspaper articles with published scientific articles on antidiabetic medicines, archived by the PubMed® database over the same study period, the same search terms was used for the PubMed® database.

Newspaper articles of any format (e.g., news article, editorial, letters to the editor) containing any reference to antidiabetic medicines or treatment, using either conventional medicines or alternative therapies (e.g., herbal or homeopathic remedies) were included in the analysis. Articles were excluded if they dealt exclusively with surgery or other procedures, or non-pharmacological therapy (e.g., diet or exercise) for diabetes, if the articles included reference to antidiabetic medicines only as part of an announcement (e.g., advertising a workshop or conference on antidiabetic medicines), if articles mentioned antidiabetic medicines in less than 10% of the article by word count (brief mention only) or if articles focusing on drug-induced diabetes or a non-diabetes indication of an antidiabetic medicine (e.g., metformin for polycystic ovary syndrome). In the case of article duplication (e.g., in the daily and Sunday equivalents), only the article with the highest word count was included in the analysis.

### Data extraction and coding frame

Taking into account the previous published literature utilising systematic content analysis, and discussions within the research team, a standardised coding frame was developed ahead of the main analyses. A pilot exercise was conducted by coding 10 articles to allow fine tuning. The final coding framework contained three main sections, as outlined in Table 1.

**Table 1:** Summary of final coding framework

Sections	Description
Structure	Bibliographic details: name of the newspaper, date of publication, headline, author, section of newspaper
Article content	Information contained within each article such as main theme regarding antidiabetic medicines, other themes covered, key perspective, class of antidiabetic medicines, benefit and harm of antidiabetic medicines, and main source of information
Judgement and rating	Subjective variable related to antidiabetic medicines information presented including the article slant, the main claim and the quality of information

If the newspaper article was linked to a scientific journal article, a fourth section was applied as follows: the scientific journal article was obtained using the author name, author affiliation and topic mentioned in the newspaper article, the content was cross checked and financial ties between authors and any pharmaceutical company were checked.

### Data collection and analysis

The coding process was carried out by two independent researchers (NS, GC). The main researcher (NS) coded the entire set of relevant articles, while the other researcher (GC) used the coding form to independently code a random sample (10%) of the articles (selected utilising random.org) to assess intercoder reliability/agreement. The level of agreement was determined by calculation of Cohen’s kappa value. Values were classified as follows: ≤ 0 poor agreement, 0.01–0.20 slight, 0.21–0.40 fair, 0.41–0.60 moderate, 0.61–0.80 substantial, and 0.81–1.00 almost perfect agreement [33].

Following data collection from all relevant articles, all data were entered into SPSS (version 25, SPSS Inc, USA) for analysis. Differences between reporting in the UK and the US were considered. The data were summarised using descriptive statistics (distribution of frequencies). The chi-square test or the Fischer exact test, as appropriate, were used to assess differences between categorical variables.

## Results

### Newspaper article selection process

The initial searches retrieved a total of 1886 UK and 798 US newspaper articles published between 1 January 2009 and 31 December 2018. From this selection of articles, 369 newspaper articles from the UK and 191 from the US satisfied study inclusion/exclusion criteria, resulting in a total of 560 newspaper articles included in the main analysis.

### Inter-coder agreement

The intercoder observed agreement and kappa values for the two coders ranged from 0.60 to 0.93 (Table 2). The agreement level between the two researchers, therefore, ranged from moderate agreement to almost perfect agreement.

**Table 2:** Observed agreement and kappa value for items coded within the coding frame (*n* = 56)

Question (number of choices)	Observed agreement (%)	Kappa value
Main themes (8 choices)	91.1	0.88
Key perspectives (9 choices)	83.9	0.77
Benefit stated (yes/no)	94.6	0.88
Risk stated (yes/no)	96.4	0.93
Main source of information (7 choices)	75.0	0.67
Main voice (7 choices)	83.9	0.77
Quotation stated (yes/no)	91.1	0.82
Article slant (4 choices)	85.7	0.80
Main claim (3 choices)	75.0	0.60
Quality of information (3 choices)	83.9	0.75

### Article frequency

**Fig. 2:**
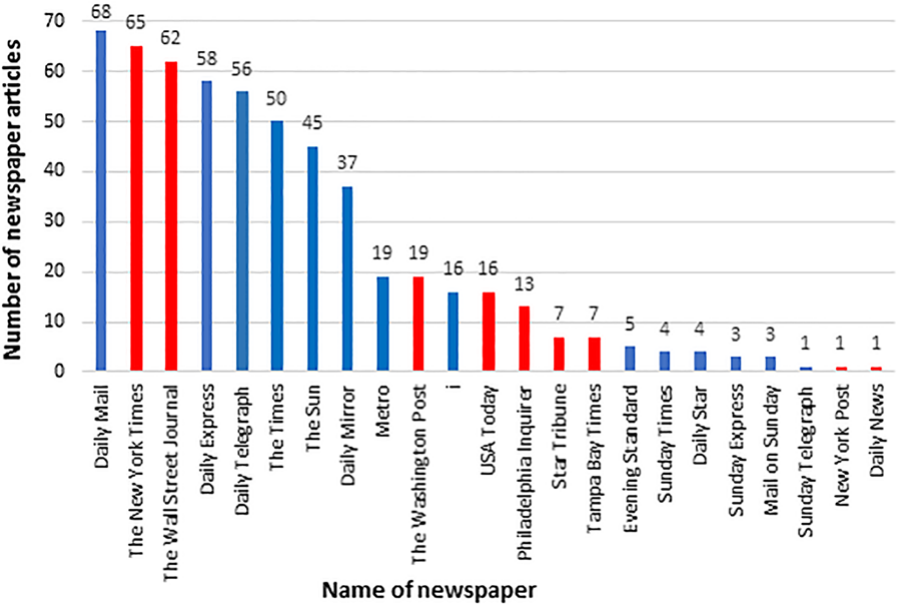
Frequency of articles about antidiabetic medicines in UK and US newspapers (2009 to 2018)

The UK newspapers which most frequently contained articles relating to antidiabetic medicines were *the Daily Mail* and *the Daily Express*, while in the US, antidiabetic medicines were reported most frequently in *The New York Times* and *The Wall Street Journal* (Fig. 2). There were no articles relating to antidiabetic medicines reported in *The Sunday Mirror* (UK), *Daily Star Sunday* (UK), *The Sun on Sunday* (UK) and *the Los Angeles Times* (US).

**Fig. 3:**
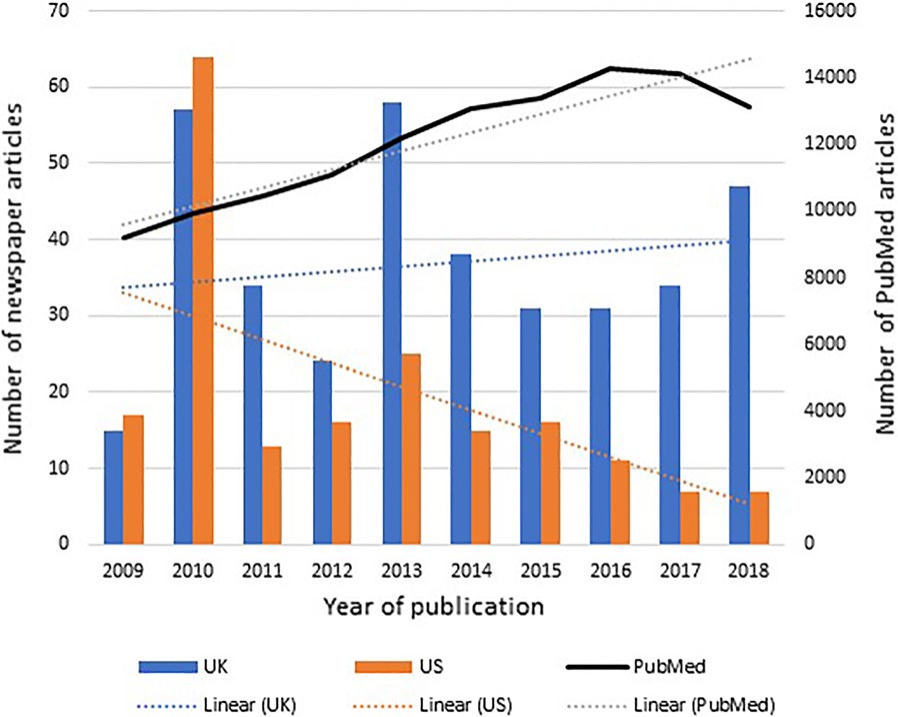
Number of articles about antidiabetic medicines in UK and US newspapers (left axis) and in PubMed articles (right axis) (2009 to 2018)

In the UK, the number of relevant articles varied considerably from year to year and showed a slightly increasing trend over the study period. In the US, article numbers declined over the study period. The corresponding number of research articles relating to antidiabetic medicines retrieved from the PubMed® database increased over the same study period is presented in Fig. 3.

### Article content

**Fig. 4:**
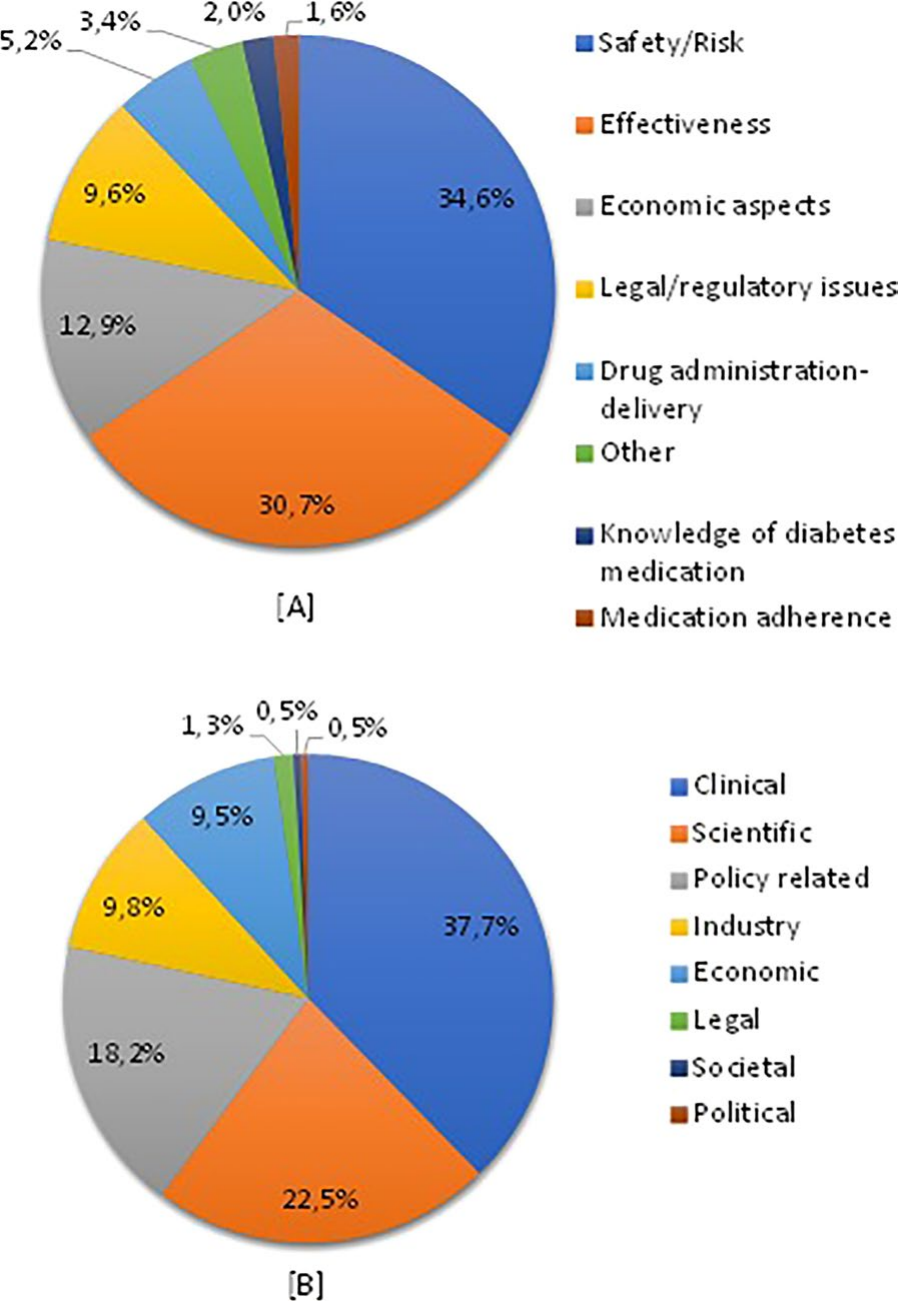
Distribution of main themes (**A**) and key perspectives (**B**) covered in the antidiabetic medicines articles (*n* = 560)

Within the 560 articles selected, the most frequently addressed main themes were safety/risk (34.6%), effectiveness (30.7%) and economic aspects (12.9%) (Fig. 4A). Articles were most frequently written from a clinical perspective (37.7%) (Fig. 4B).

Besides safety/risk, effectiveness and economic aspects, other examples of topics included were (i) approval of new antidiabetic medicine (dapagliflozin) by the US Food and Drug Administration; (ii) new routes of insulin delivery, i.e., tablet/pill and inhaler; and (iii) medication non-adherence in diabetes patients commonly related to metformin and DPP4 inhibitor use due to side-effects (diarrhoea, flatulence). The UK and the US demonstrated somewhat different profiles regarding main themes; effectiveness was the most frequent theme in the UK (39.3%), while almost half (48.7%) of US articles had a main theme of safety/risk (Table 3).

**Table 3:** Main themes regarding antidiabetic medicines covered in the UK and US newspapers

Main theme	UK (*n* = 369)		US (*n* = 191)		*p* value*
	Frequency	%	Frequency	%	
Safety/risk	101	27.4	93	48.7	< 0.001
Effectiveness (%)	145	39.3	27	14.1	< 0.001
Economic aspects	49	13.3	23	12.0	0.678
Legal/regulatory issues	25	6.8	29	15.2	0.001
Drug administration-delivery	24	6.5	5	2.6	0.049
Other	12	3.3	7	3.7	0.798
Knowledge	6	1.6	5	2.6	0.423
Medication adherence	7	1.9	2	1.0	0.448

*Chi square test

**Fig. 5:**
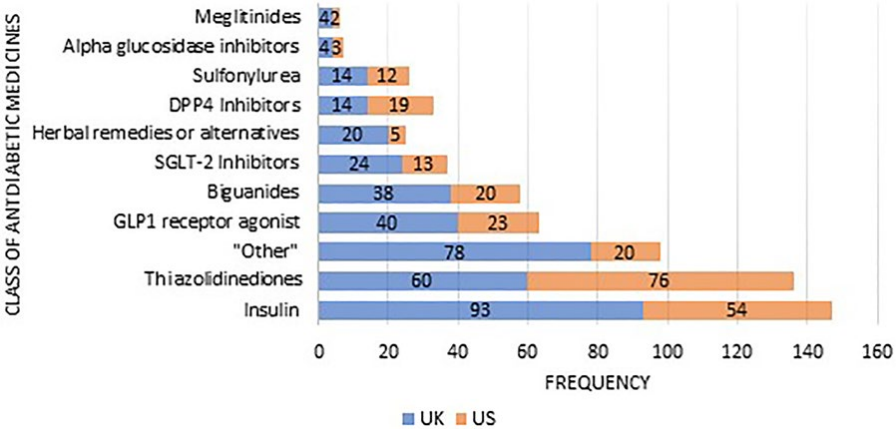
Diabetic medication classes specifically mentioned in published articles (*n* = 636)

A total of 500 (89.3%) of the articles included in the main review specified the class of antidiabetic medicines being considered. Insulin (*n* = 147; 23.1%) and thiazolidinediones (*n* = 136; 21.4%) were the most commonly mentioned classes of antidiabetic medicines. The proportions differed between the UK and USA as outlined in Fig. 5, most markedly in relation to “other” classes of medications which were much more frequently discussed within UK newspaper articles (20.1% vs 8.1%) and thiazolidinediones which were much more frequently discussed within US newspaper articles (15.4% vs 30.8%). These latter differences reached statistical significance (*p* < 0.002 and *p* < 0.001, respectively).

The benefits of antidiabetic medicines were specified in over half (*n* = 307; 54.8%) of the articles included in the main review. In these articles, the most frequently reported benefit of antidiabetic medicines related to control of blood sugar (53.1%) followed by prevention or treatment of diabetes complications (17.8%) and reduction in weight and/or appetite (17.1%). The frequency of reporting benefits of antidiabetic medicines was higher in UK newspapers (59.1%) than in US newspapers (46.6%; *p* = 0.005). The more frequent discussion of “other” classes of medication within UK newspapers partly influenced this higher trend.

It is clear from the published scientific literature that diet and exercise (with resultant weight loss) are very important aspects of diabetic management which should be implemented alongside pharmacotherapy. The importance of combining diet and exercise with antidiabetic medicines was, however, stated in only 5.5% and 4.6% of UK and US articles, respectively.

**Fig. 6:**
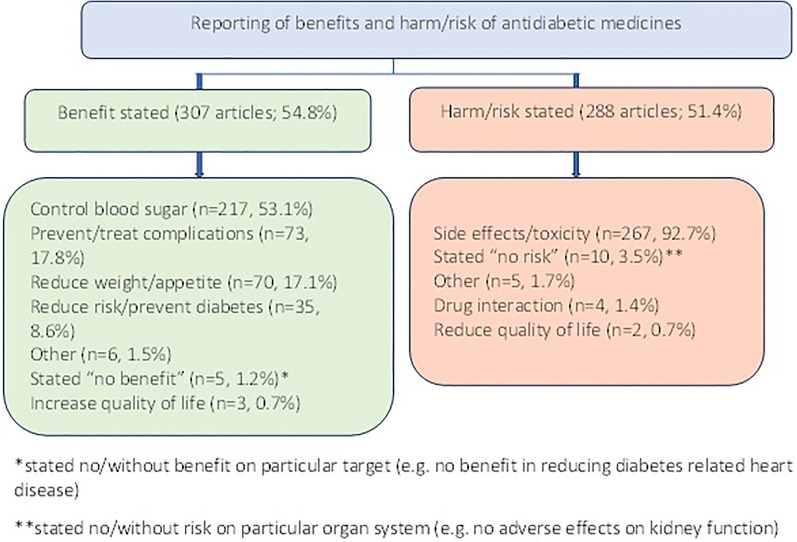
Summary of benefits and risks of antidiabetic medicines

The harms and risks associated with antidiabetic medicines were specified in approximately half (*n* = 288; 51.4%) of included articles. Frequency of reporting harms and risks was higher in US newspaper articles (61.3%) than in UK newspapers (46.3%; *p* = 0.001). A summary of benefits and harms/risks of antidiabetic medicines addressed is presented in Fig. 6.

**Fig. 7:**
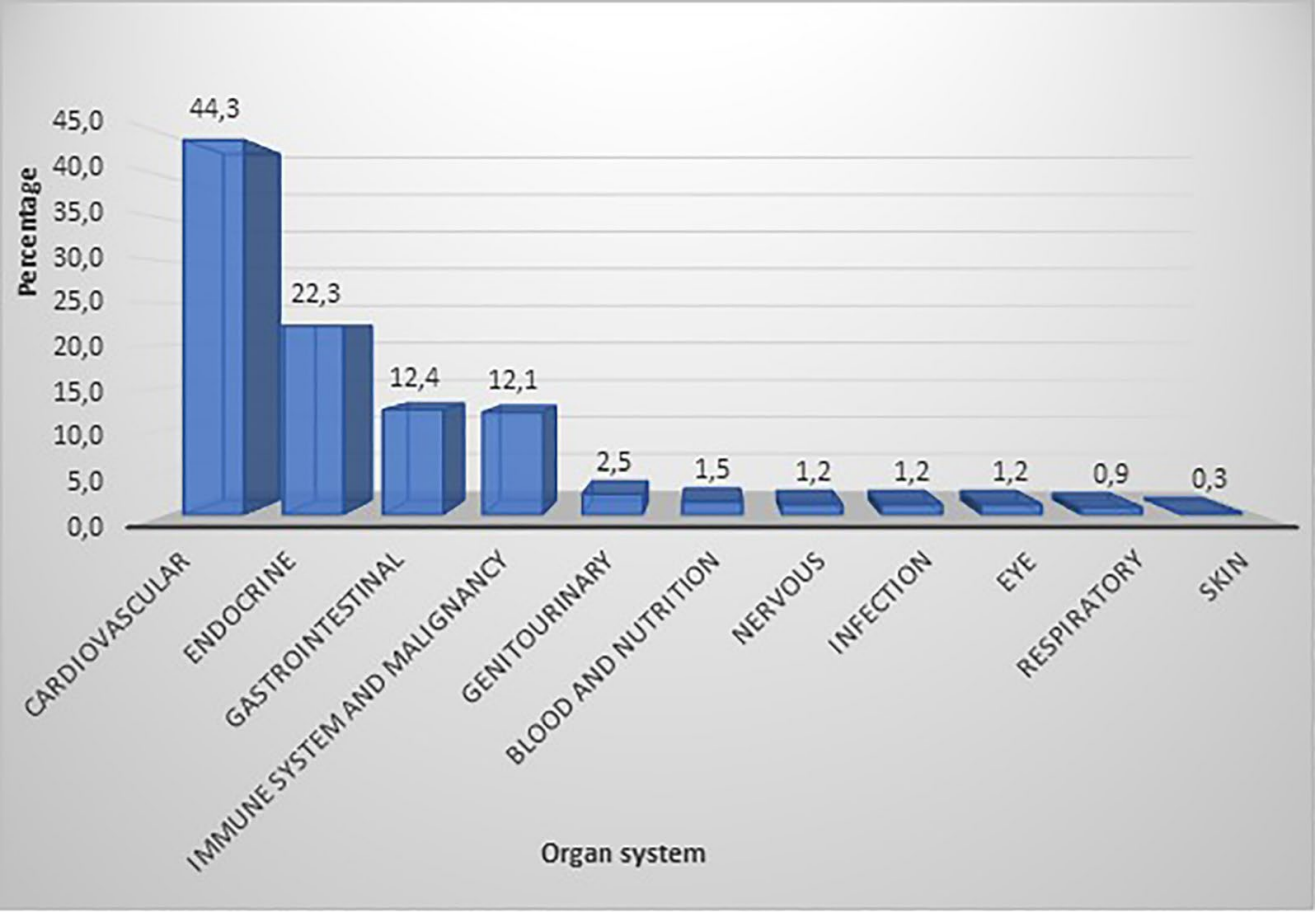
Organ systems reported as being harmed by antidiabetic medicines

Harm/risk reported as being caused by antidiabetic medicines was classified into organ systems according to the relevant chapter of the British National Formulary 76th edition [34]. The most frequently reported organ systems harmed by antidiabetic medicines were the cardiovascular, endocrine and gastrointestinal systems (Fig. 7).

The most frequently specified adverse effects within the cardiovascular system were heart attack and stroke. These were mainly (81.8%) linked to rosiglitazone from the thiazolidinediones class. Hypoglycaemia was the main adverse effect within the endocrine system, and this was mainly linked to the use of insulin. The most frequently reported gastrointestinal adverse effects were diarrhoea, nausea, flatulence, vomiting and loss of appetite. Metformin was the most frequently implicated medicine regarding gastrointestinal side effects. Regarding immune system/malignancy, pioglitazone and GLP-1 drugs (exenatide and liraglutide) were reported to cause bladder and pancreatic cancers, respectively. Cardiovascular and immune system/malignancy risks associated with antidiabetic medicines were more likely to be stated in US newspaper articles (55.2% and 13.8%, respectively) than in UK newspapers (35.4%; *p* < 0.001 and 10.7%; *p* = 0.019, respectively).

Most of the qualifying articles reported that the information source regarding antidiabetic medicines was a scientific journal/paper/report or its authors (49.1%) or a government report (30.0%). The remainder cited health or pharmaceutical industry (8.1%) or other sources including patient and patient’s relative (5.3%), healthcare professional (4.6%), scientist (2.0%) and scientific meetings (0.9%).

Of the 112 articles which cited a specific scientific journal as their source of information, 12 reported that the scientists involved had financial ties with the pharmaceutical company. Using the author name, author affiliation and topic mentioned in the newspaper articles, it was possible to retrieve a total of 85 the latter scientific articles, 29 of which stated financial ties between the author and pharmaceutical company.

Scientists, researchers or academics were found to provide the main voice for more than a third (36.6%) of the articles, while the main voice for the remainder of articles were government, e.g., National Health Service, European Medicines Agency, or Food and Drug Administration (25.2%), the pharmaceutical industry (15.5%), healthcare professionals (10.2%), major charities (3.4%) and journalists (2.5%). More than half of the articles (56.3%) included quotations from scientists/researchers/academics, governments, major charities, healthcare professionals, industry spokespersons and patients.

Just under two-fifths (39.5%) of articles were classified as having a negative slant [35] on antidiabetic medicines, and a slightly smaller proportion (34.6%) of the articles were written using a positive slant. A neutral slant was found in 16.4% of articles, and mixed positive and negative approaches, for example, portraying the benefits and risks of antidiabetic medicines, were reported in approximately one-in-ten articles (9.5%). Articles in UK newspapers informed readers of benefits of antidiabetic medicines more often than US newspapers (43.4% vs 17.8%), and risks of antidiabetic medicines less often (31.7% vs 54.5%) (*p* < 0.001 in both cases).

The majority of relevant articles (91.8%) were judged as having a balanced judgement, while 8.2% of the articles were rated as having an exaggerated judgement; no article was judged as having understated judgements. The number of articles with exaggerated judgement was significantly higher in UK newspaper articles (11.1%) than in the US (2.6%; *p* < 0.001). An example of an exaggerated judgement was as follows *“A new three-in-one “super drug” to cure diabetes and obesity could soon help to save lives”* (*The Daily Express*, 9 December 2014).

The quality of antidiabetic medicine information presented in every article was scored between 1 and 10 (1 representing the lowest and 10 representing the highest quality). Overall, just over one in five (21.4%) of the articles were scored as having excellent quality of information (scored 8–10), just under half (47.7%) as having average quality information (scored 4–7) and almost one-third (30.9%) as having poor quality information (scored 1–3) regarding antidiabetic medicines. An article categorised as poor quality generally only contained limited information about antidiabetic medicine(s) with no or poor evidence or a lack of balanced judgement. Attributes of an excellent quality article included the provision of highly relevant information about antidiabetic medicines, for example, benefits and risks of antidiabetic medicines, had balanced judgement, was based on evidence, provided sources of information to support its claims and included quotations from experts or other relevant spokespersons. The frequency of articles reporting excellent quality of information was significantly higher in US newspapers (33.5%) compared with UK publications (15.2%; *p* < 0.001).

## Discussion

The trend of increasing articles over time in the UK newspapers aligned with the PubMed trend, but this was not the case for the US newspapers. In both jurisdictions, only a small fraction of published scientific articles gained media attention. Since press releases describing the content of scientific articles to journalists play an important role in uptake of articles by newspapers [36, 37], scientific authors should be more proactive in providing press releases describing their main findings and their relevance to patient care. It is clear from the present results that there is considerable scope to enhance public access, via newspaper articles, to a very large scientific literature on antidiabetic medicines.

Safety or risk, together with effectiveness and economic aspects of antidiabetic medicines, were the most frequently covered topics in the relevant newspaper articles in the present research. More than a third of all articles covered safety or risks associated with antidiabetic medicines, the majority of which were written from the clinical perspective and focused on medication side effects. A number of articles reported particular diabetes medicines with very serious side effects, for example, pioglitazone dramatically increasing the risk of bladder cancer when used every day for more than 2 years, or the association of rosiglitazone with heart attack and heart failure. Unfortunately, a number of newspaper articles reported these side effects without adding further advice for patients who were already receiving these medicines. Those articles tended to report the findings of trials without involving healthcare professionals or other sources of information. Conversely, some newspaper articles correctly advised patients not to stop taking the medicine, and not to change or lower the dose without speaking to their doctor to discuss an alternative. The focus on the risks of antidiabetic medicines, particularly if sensationalised (lack of balanced judgement), could precipitate anxiety or major concerns for patients regarding their medication that could lead to medication non-adherence [38–40], or to patients seeking an unnecessary change to their prescribed medication. Interestingly, the publication of risks relating to other medications in the mass media (for example, calcium channel blockers [41] and hormone therapy [42–44], has indeed resulted in a substantial decline in the use of these medications.

The frequency of reporting harms and risks of antidiabetic medicines in US newspapers was greater than 60%, which was significantly higher than in UK newspapers (46%) in the present research. This was also higher than the frequency of harm reported in a previous study which investigated US news media coverage of pravastatin, aspirin and alendronate, in which potential harm of these medications was reported in 47% of articles [45].

The most frequently reported side effects of antidiabetic medicines in the present study involved the cardiovascular, endocrine and gastrointestinal systems. These were frequently associated with thiazolidinediones, insulin and metformin, respectively. These findings reflect published medical literature in which a high concern regarding cardiovascular risks with the use of rosiglitazone has been reported [46–49]. Furthermore, there has been a general reluctance from patients to receive insulin or intensify their insulin therapy because of the fear of hypoglycaemia [47, 50]. However, the newspaper articles did report the essential role of insulin for diabetes treatment and introduced readers to new dosage forms and novel insulin analogues that minimise the risk of hypoglycaemia. Furthermore, a number of articles also provided advice for patients on action to take in the event of hypoglycaemia. Metformin is well recognised as the main agent causing gastrointestinal disturbances, an adverse consequence which is seen in almost 30% of patients taking this medicine [47, 48, 51]. Usefully, a number of newspaper articles reported that switching from an immediate-release tablet to a long-acting or modified-release form of metformin could help solve the gastrointestinal disturbance.

Discussion of the effectiveness and benefits of antidiabetic medicines in the newspaper articles included discussion not only of the ability of the medication to control blood sugar levels. Other benefits discussed included a reduction of diabetes complications, a reduced risk of developing diabetes in pre-diabetic patients, increased quality of life, and other benefits, such as improved survival time and reduced hospitalisation. Some articles also outlined benefits which can be obtained from other classes of medication: for example, lorcaserin (a weight-loss medicine) that reduces the risk of diabetes in pre-diabetes patients; leflunomide (anti-rheumatic medicine) that lowers blood glucose levels and reverses insulin resistance; dextromethorphan (cough suppressant) that can increase insulin levels and enhance glucose tolerance; sucralfate (gastroprotective and chelator agent) that can reduce the amount of sugar and fat absorbed from food; otelixizumab (monoclonal antibody) that can halt or dramatically reduce the need for insulin injections among newly diagnosed type 1 diabetics; and ranibizumab (monoclonal antibody) that can treat proliferative diabetic retinopathy.

A number of herbal remedies or alternative medicines were also reported to prevent or treat diabetes including cinnamon, turmeric, vinegar, milk thistle and cannabis. These articles were mainly based on limited or no objective evidence and as a consequence, readers could be substantially misinformed. As pointed out by Bubela et al. [52], in their content analysis on UK, US, Australia, New Zealand and Canadian newspapers, the negative results and risks of herbal remedies are only partly reported in newspaper articles.

A scientific journal/paper/report or its author was the most frequently cited source of information regarding antidiabetic medicines in the current research. However, the information presented in the scientific journal was not reported or critiqued sufficiently in some articles to provide the reader with a full insight into the findings. Lack of adequate scientific evidence to support health advice has been deemed to be a serious issue in health journalism internationally, for example, in Australia [53] and the UK [54]. Maksimainen [55] emphasised that if news media cited a research paper as their source of information, a rigorous review of information presented in the research paper was required before delivering the facts to the reader. This does not always happen, for example, a study focusing on the US news media coverage of pravastatin, alendronate and aspirin found that it did not include adequate or complete information regarding the benefits and risks of these medications [45].

In the present study, there were 112 articles which cited a specific scientific journal as their source of information regarding antidiabetic medicines, with 29 of the scientific papers citing a conflict of interest. This conflict, for example financial ties between author and medicine manufacturer was often not reported. Others have found that such financial ties are generally not mentioned in newspaper articles [45, 52], highlighting the fact that the news media pay little attention to a factor that could potentially lead to biased reporting [38].

The majority of relevant articles in the present study were classified as having balanced judgement, where the main message was not exaggerated or understated. This aligns with other healthcare studies in the UK and/or US newspapers, for example, reporting on healthcare applications [56], pharmacogenetics [57] and online health information [58]. In the present study, the number of articles with exaggerated judgement was, however, higher in the UK than in the US. This was mainly because the UK newspapers were more likely to claim the medication as a “cure” for diabetes, as a “wonder pill” or “without side effects”. Some articles claimed the medication could treat diabetes based only on pre-clinical animal studies. If the benefits of a particular medication are overstated or exaggerated, patients or the public could have their expectations unrealistically raised regarding a cure for their disease or improvement of their health [59].

The reporting quality about antidiabetic medicines in newspapers in the present research was overall classified as average, but a third of the total articles was deemed to present poor quality information. As highlighted by Wilson et al. [53] as well as by Wang and Lai [60], the quality of reporting medical information in the mass media, including newspapers, is often poor. The quality of press releases issued by scientific journals or article authors plays an important role in the quality of information reported in newspapers; the higher the quality of press releases, the higher the quality of articles in newspapers [61, 62]. Improvement is, therefore, required in the quality of press releases by scientific journals or authors.

With the wide range of topics about antidiabetic medicines covered in the newspaper articles, it is likely that views and beliefs of diabetic patients regarding antidiabetic medicines may be influenced both positively and negatively. Positive influences of mass media have been reported, including improvement in patient understanding of their health condition(s) and improved patient behaviour to achieve a better state of health [25–30, 63, 64]. However, negative influences have also been reported in an online survey of patients who had been prescribed an oral anticoagulant in the US. Patients who were exposed more frequently to health information via the mass media had a tendency to have less necessity beliefs (implicit judgements of personal need for the treatment) regarding their medication and were less adherent to their medication [65]. As described earlier, healthcare practitioners have two relationship models with the health media; they may use the media to influence patients, but they also have the challenging responsibility to counteract media articles which promote harmful or unhealthy lifestyle or practices [66].

## Limitations

This present study has several limitations. Antidiabetic medicines reported in other mass media sources (e.g., television and radio) were not covered in this study. However, newspaper article content has a close correlation with the subject material covered by other media [17–19]. The following limitations are related to The Nexis® UK database: (i) several highly circulated newspapers, for example, *Newsday*, *Chicago Tribune* and *The Boston Globe*, were not available thus were not included in the study; (ii) the Nexis® UK database only provides articles published in *the Los Angeles Times* for the previous 6 months; (iii) only abstract versions of articles published in *the Wall Street Journal* were accessible.

## Conclusions

This study has revealed that antidiabetic medicines are reported on by UK and US newspapers. As media portrayal has the potential to negatively or positively influence patients’ views of their medication, it is recommended that healthcare professionals ascertain patients’ views about their medicines, proactively provide objective, balanced information and promote medication adherence. The quality of reporting on antidiabetic medicines in newspapers in the present study was deemed to be poor quality in approximately a third of the total articles examined. Since many of the newspaper articles were based on scientific papers, scientific authors should ensure that press releases are issued alongside their published papers in order that journalists receive a lay language version of the work that can be used in the development of their articles. It would be hoped that such articles would be more objective and less sensational.

## Data Availability

The data used to support the findings of this study are included in this published article.
